# Geometrically-controlled polarisation processing in femtosecond-laser-written photonic circuits

**DOI:** 10.1038/s41598-017-09462-0

**Published:** 2017-09-12

**Authors:** Ioannis Pitsios, Farid Samara, Giacomo Corrielli, Andrea Crespi, Roberto Osellame

**Affiliations:** 1 Istituto di Fotonica e Nanotecnologie - Consiglio Nazionale delle Ricerche (IFN-CNR), p.za Leonardo da Vinci 32, 20133 Milano, Italy; 2 Dipartimento di Fisica - Politecnico di Milano, p.za Leonardo da Vinci 32, 20133 Milano, Italy; 3 Present Address: GAP-Quantum Technologies, Université de Genève, Chemin de Pinchat 22, Genève, 1211 Switzerland

## Abstract

Polarisation of light is a powerful and widely used degree of freedom to encode information, both in classical and quantum applications. In particular, quantum information technologies based on photons are being revolutionised by the use of integrated photonic circuits. It is therefore very important to be able to manipulate the polarisation of photons in such circuits. We experimentally demonstrate the fabrication by femtosecond laser micromachining of components such as polarisation insensitive and polarising directional couplers, operating at 1550 nm wavelength, where the two opposite behaviours are achieved just by controlling the geometric layout of the photonic circuits, being the waveguides fabricated with the same irradiation recipe. We expect to employ this approach in complex integrated photonic devices, capable of a full control of the photons polarisation for quantum cryptography, quantum computation and quantum teleportation experiments.

## Introduction

Integrated optics is a very powerful platform to produce miniaturised complex photonic devices with improved scalability, robustness and suitability for field application^1^. This approach greatly benefited classical optics communications, which is at the basis of today’s information society. A similar trend is being adopted also for quantum optical devices, fostering an exponential increase in the layout complexity of integrated photonic circuits for various quantum applications, from computation to simulation^2–6^. Integrated quantum photonics is currently based on consolidated technologies, such as silicon on insulator^3^ and silica on silicon^2^, as well as on more innovative approaches as waveguide writing by femtosecond laser micromachining (FLM)^4–6^. The two main advantages that FLM introduced in integrated quantum photonics are: the possibility to manipulate polarisation-encoded photons on-chip^5^ and unique 3D fabrication capabilities^7^. The former enables the direct on-chip transfer of many protocols already developed in quantum optics and based on polarisation encoding. The latter opens the way to innovative 3D layouts that can produce novel functionalities in more compact geometries.

The polarisation sensitivity of the FLM fabricated devices comes from the low (~10^−4^–10^−5^) optical birefringence that is typically present in this type of waveguides. Depending on the material, birefringence in FLM can originate from one or more of the following sources. It can be due to the creation of periodic nano-structures aligned orthogonally to the writing beam polarisation^8^, it can arise due to asymmetric material stresses induced in the focal volume^9^, or it may be due to ellipticity of the written waveguide cross section^10, 11^. While high birefringence is desirable to achieve effective polarisation manipulation in compact devices, on the other hand this same property induces unwanted strong delays between the two main polarisation components also during plain propagation in the chip, thus rapidly destroying the original polarisation state. The birefringence of the glass waveguides produced by FLM represents a very good compromise in this view, as it is low enough to produce an easily compensable effect on the polarisation state, even when propagating in several-centimetre-long waveguides, but it is high enough to produce a significant change in the coupling coefficients for different polarisations in a directional coupler (the integrated optics equivalent of a beam splitter).

Taking advantage of this property, polarisation dependent components such as integrated polarising beam splitters^12^ and polarisation retarders^13^ have been presented for 1550 nm wavelength in fused silica. Polarisation waveplates^14^ have been demonstrated in the same material at 800 nm wavelength, while partially polarising beam splitters^15^, waveplates^16^ and polarisation insensitive directional couplers^17^ have been successfully demonstrated in alumino-borosilicate glasses at 785 nm wavelength. As it can be seen, scattered results in terms of material and wavelength are present in the literature. Here, we present a comprehensive study of polarisation sensitive and insensitive components produced by FLM in what are, in our opinion, very interesting conditions in terms of material and wavelength: the alumino-borosilicate glass EAGLE (Corning) and 1550 nm operating wavelength. The glass used in this work is specifically EAGLE 2000, which is currently out of production, being substituted by the new version EAGLE XG (however, as far as the waveguide writing process by FLM is concerned, we have found no difference in the use of the two glasses). On the one hand, the use of EAGLE glass allows producing high-refractive-index-change waveguides at very high writing speeds, in the order of several cm/s. On the other hand, the choice of 1550 nm wavelength allows one to take advantage of the very low losses in fiber transmission, with interesting applications to quantum optical communication^18^, distributed quantum computing^19^ and quantum teleportation^20^ experiments. In particular, we demonstrate a new approach to achieve polarisation insensitive directional couplers, we demonstrate high-performance polarising directional couplers and we show that also rotated waveplates can be produced in the same conditions. Finally, it should be noted that all these components are manufactured with the same fabrication recipe, this is very important in future complex photonic devices where it will be possible to combine polarisation sensitive and insensitive components on the same chip by only playing with their geometrical layout.

## Results

### Directional couplers formalism

**Figure 1:**
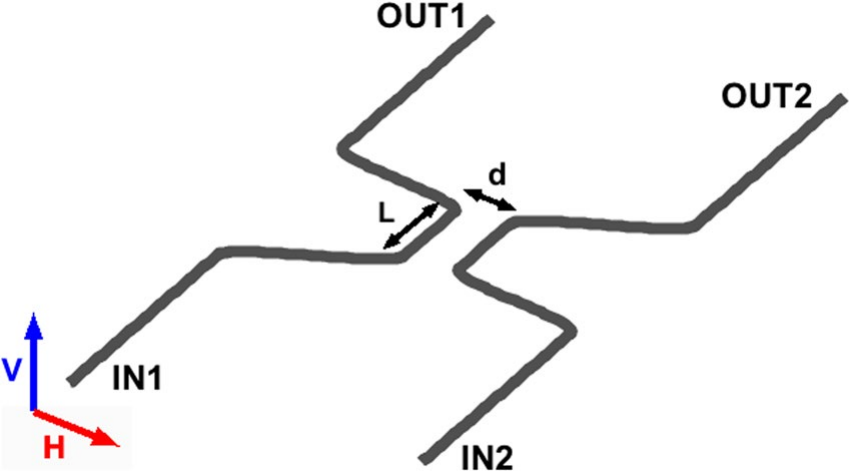
Typical geometry of a directional coupler, lying on the horizontal plane. Two waveguides are brought close at a distance *d* for a length *L*. Two input ports IN1 or IN2 and two output ports OUT1 and OUT2 can be identified. Orientations of horizontally (H) and vertically (V) polarisations with respect to the coupler plane are indicated.

A directional coupler (DC) is the integrated-optics equivalent of the bulk-optics beam splitter. It consists of two waveguides that become proximal at a separation *d* for a length *L* (Fig. 1). Within such a region, the two waveguides interact via evanescent-field coupling^21, 22^. Light injected in either of the input ports (IN1 or IN2) thus partly remains in the same waveguide (BAR), while partly is transferred to the other arm (CROSS). In analogy with the bulk beam splitter we can define power transmission (*T*) and reflection (*R*) coefficients for a DC as:

1 $$T=\frac{{P}_{{\rm{CROSS}}}}{{P}_{{\rm{CROSS}}}+{P}_{{\rm{BAR}}}}\quad R=\frac{{P}_{{\rm{BAR}}}}{{P}_{{\rm{CROSS}}}+{P}_{{\rm{BAR}}}}$$

where *P*_BAR_ and *P*_CROSS_ are the output powers on the two arms.

If device losses are negligible or uniformly distributed along the waveguides, *T* and *R* do not depend on the choice of the input and oscillate as a function of the interaction length *L* according to ref. 21:

2 $$\begin{array}{c}T=\frac{{\kappa }^{2}}{{\sigma }^{2}}\,{\sin }^{2}\,(\sigma L+{\varphi }_{0}),\\ R=1-T=\frac{{{\rm{\Delta }}}^{2}}{4{\sigma }^{2}}+\frac{{\kappa }^{2}}{{\sigma }^{2}}\,{\cos }^{2}\,(\sigma L+{\varphi }_{0})\end{array}$$

with the angular frequency *σ* being defined as *σ* ^2^ = *κ* ^2^ + Δ^2^/4. In these expressions *κ* is the coupling coefficient between the optical modes, Δ is their detuning in propagation constant, and *ϕ*_0_ takes into account coupling occurring in the curved waveguide segments incoming and departing from the interaction region.

Of course, if waveguides are identical Δ = 0 and Eq. (2) simplify to:

3 $$\begin{array}{c}T={\sin }^{2}\,(\kappa L+{\varphi }_{0}),\\ R={\cos }^{2}\,(\kappa L+{\varphi }_{0})\end{array}$$

The coupling coefficient *κ* depends on the overlap integral between the two waveguide modes, and thus it depends on separation *d* between the two waveguides. For weakly guiding, femtosecond laser written waveguides, such dependency is well approximated by an exponential function^23^ of the kind:

4 $$\kappa ={\kappa }_{0}{e}^{-\frac{d}{{d}_{0}}}$$

where *κ*_0_ and *d*_0_ are proper constants. Evidently, by manipulating the interaction length and the separation of the waveguides, one can fully tune the coupler splitting ratio from null to unitary transmissivity or reflectivity.

### Polarisation-splitting directional couplers

A polarisation-splitting directional coupler (PDC) is the integrated equivalent of a bulk polarising beam splitter. The function of such a device is to separate orthogonal polarisations into different output arms. For instance, if vertically polarised light is coupled in port IN1 and all of it exits the device at OUT1, then horizontally polarised light injected in port IN1 should exit all from OUT2. As global figures of merit for the quality of a PDC we can define the logarithmic extinction ratios in transmission and reflection:

5 $${{\rm{ER}}}_{T}=|10\,{\mathrm{log}}_{10}\,\frac{{T}_{V}}{{T}_{H}}|\quad {{\rm{ER}}}_{R}=|10\,{\mathrm{log}}_{10}\,\frac{{R}_{V}}{{R}_{H}}|$$

expressed in decibels. An ideal device would have infinite extinction ratios both in transmission and in reflection.

PDCs can be realized exploiting waveguide modal birefringence, as shown in the literature, which reports partially polarising PDCs in borosilicate glass, working at 800 nm wavelength^15^, and fully polarising PDCs in fused silica, working at 1550 nm wavelength^12^. Here, as a first component of our polarisation manipulation platform, we address the optimisation of PDCs in borosilicate glass working in the 1550 nm wavelength range.

The operation principle of a PDCs is simple. In general, because of waveguide birefringence, the power oscillation between the two waveguides in the DCs, described by (3), presents a different periodicity for different polarisation states (see the Methods for a more formal description of the polarisation behaviour of a generic two-port device). While for shorter interaction lengths *L* the difference in splitting ratio may be minimal, the polarisation dependence may become larger with increasing *L* where the two beatings accumulate larger phase difference. In particular, for a proper choice of the DC geometrical and coupling parameters, one may reach a point in which the two oscillations are in anti-phase, which is indeed the condition for a PDC. From equation (3), and assuming for simplicity *ϕ* = 0, we can write mathematically the PDC condition as follows:

6 $$\{\begin{array}{l}{\kappa }_{V}\,L=m\pi \quad \vee \quad {\kappa }_{V}\,L=(m+\frac{1}{2})\pi \\ {\kappa }_{H}\,L={\kappa }_{V}\,L\pm \frac{\pi }{2}\end{array}$$

where *m* is integer and we have differentiated *κ*_*V*_ and *κ*_*H*_ as coupling coefficients for two orthogonal polarisations, here considered vertical (V) and horizontal (H). The choice of the first equation (with or without the $\tfrac{1}{2}\pi$ term) depends on the desired behaviour for the V polarisation, namely if one wants it totally reflected or totally transmitted. The sign in the second equation will be plus (minus) if *κ*_*V*_ is smaller (larger) than *κ*_*H*_. It is not difficult to observe that satisfying exactly these equations requires *κ*_*H*_/*κ*_*V*_ to be a ratio of integer numbers. Namely, (6) can be elaborated as follows:

7 $$\{\begin{array}{ccc}\frac{{\kappa }_{H}}{{\kappa }_{V}} & = & \frac{2m+1}{2m}\quad \vee \quad \frac{{\kappa }_{H}}{{\kappa }_{V}}=\frac{2m+2}{2m+1}\\ \,\,\,L & = & \frac{\pi }{2({\kappa }_{H}-{\kappa }_{V})}\end{array}$$

where we have assumed *κ*_*H*_ > *κ*_*V*_. In principle the ratio *κ*_*V*_/*κ*_*H*_ could be modulated continuously by the choice of the interaction distance *d*, and it should be possible to fulfill (7) up to an arbitrary high precision by a careful optimization of both *d* and *L*. In practice, the non-perfect reproducibility of the fabrication technology would make this process uneffective. Therefore, as it will be discussed later in detail, we will choose a value for *d* sufficiently close to the optimum, and then we will proceed in optimizing *L*, concentrating in particular on one of the two extinction ratios (either ER_*T*_ or ER_*R*_), thus sligthly compromising on the other one.

**Figure 2:**
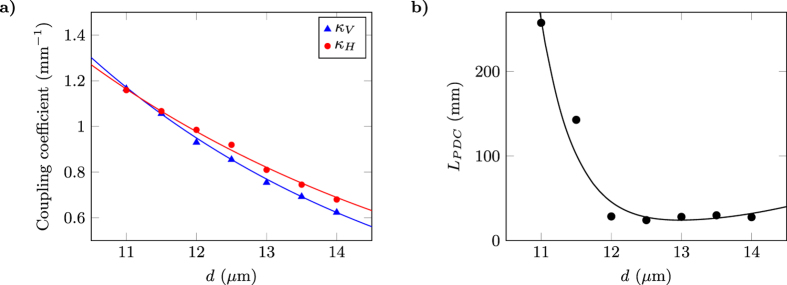
(**a**) Experimentally measured coupling coefficients *κ*_*H*_ (red circles) and *κ*_*V*_ (blue triangles) for H and V polarised light respectively, as a function of the waveguide separation *d* in the DCs. Solid lines are exponential fits. (**b**) Predicted interaction lengths *L* for achieving a PDC, as a function of *d*, obtained by substituting in the second equation of (7) the experimental values of *κ*_*H*_ and *κ*_*V*_ (black circles) and their exponential fits (solid line). Error bars are smaller or comparable to the marker size.

Our femtosecond laser written waveguides in borosilicate glass, supporting a single mode at 1550 nm wavelength, yield quite a low modal birefringence *b* = 3.6 · 10^−5^ (see the Methods section for details on the fabrication and characterisation process). The birefringence axis is oriented vertically, due to the symmetry constraints of the waveguide writing process^16^. This gives distinct guided modes for H and V polarisations, with a slightly different size and shape. The measured H mode size (1/*e* ^2^) is 15.6 × 15.1 *μ*m^2^ while the V mode size is 15.7 × 15.5 *μ*m^2^. A mode-size difference results in different *κ*_0_ and *d*_0_ values in (4) depending on the light polarisation, which produce in general a polarisation dependent *κ* value for a given interaction distance *d* of a directional coupler. As a first step to the realisation of a PDC, we characterised thoroughly the dependence of the coupling coefficients *κ*_*H*_ and *κ*_*V*_ on the interaction distance *d*. To this purpose, we fabricated DCs with the same length and separations varying from 11 *μ*m to 14 *μ*m. The output power distribution for each polarisation was collected and the different coupling coefficients were retrieved by inverting relations (3). Results are reported in Fig. 2a.

An estimate of the interaction length *L*_*PDC*_ required to achieve a PDC can be given by simply substituting the measured values of *κ*_*H*_ and *κ*_*V*_, as a function of *d*, in the second equation of the condition (7) (see Fig. 2b). It is important to note that *L*_*PDC*_ becomes large both when *d* is too short and the coupling coefficients for the two polarisations tend to be similar, and when *d* is too large and both coupling coefficients become small (even though they progressively differentiate). Thus, there is an optimum value for *d* which gives the shortest device. According to the experimental data and their best fit, in our case such value should be around *d*_*opt*_ = 12.5 *μ*m, corresponding to a *L*_*PDC*_ ~ 30 mm.

**Figure 3:**
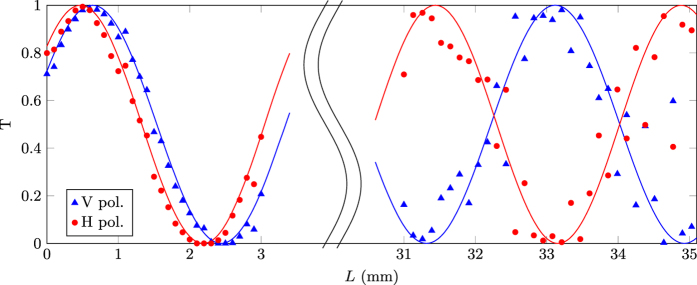
Experimental measurements of the transmission *T* of DCs with different interaction length *L* and waveguide separation *d* = 12.5 *μ*m, for H (red circles) and V (blue triangles) polarised input light. Solid lines are best-fitting theoretical curves according to Eq. (3). Error bars are smaller or comparable to the marker size.

Once the optimum interaction distance *d*_*opt*_ was chosen, in order to measure more accurately *κ*_*H*_, *κ*_*V*_, *ϕ*_*H*_ and *ϕ*_*V*_ for that distance, and to have a better estimate of the actual *L*_*PDC*_, we fabricated 31 DCs spanning *L* = 0–3 mm. The left part of the graph in Fig. 3 reports the measured transmission *T* for these devices, for the two polarisations as a function of *L*. By extrapolating the sinusoidal trends to longer lengths we identified ${L}_{PDC}\simeq 33\,{\rm{mm}}$, in agreement with the previous estimate. Subsequently, we fabricated 40 devices to scan *L* = 31–35 mm. Measured transmission values for these devices are reported in the right part of the graph in Fig. 3.

**Figure 4:**
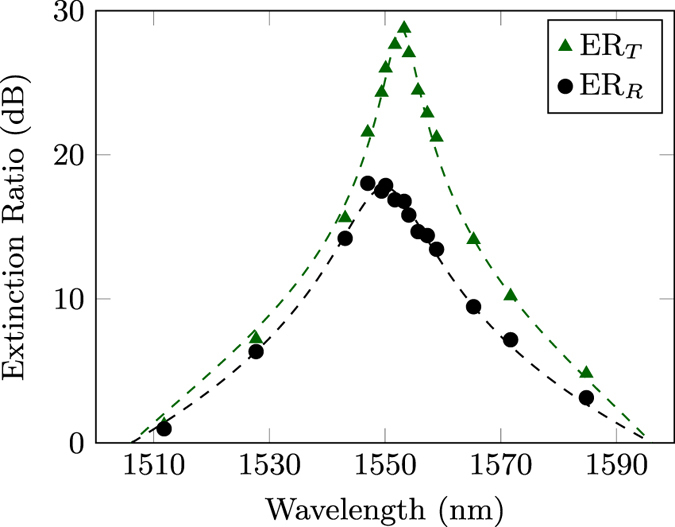
Spectral characterisation of the transmission and reflection extinction ratios for a PDC fabricated with geometrical parameters *d* = 12.5 *μ*m and *L* = 33.2 mm. Dashed lines are best-fitting curves from an analytic model in which we assumed a linear dependency of the coupling coefficients *κ*_*H*_ and *κ*_*V*_ from the wavelength and *T*_*V*_ and *T*_*H*_ can oscillate with a generic non-unitary visibility. Error bars are smaller or comparable to the marker size.

The device showing the best performance was the one with *L* = 33.2 mm, which yielded transmissions *T*_*V*_ = 0.980 and *T*_*H*_ = 0.002 for the two polarisations respectively, corresponding to ER_*T*_ = 26.9 dB and ER_*R*_ = 17 dB at the design wavelength of 1550 nm; this device was chosen for an in-depth characterisation. Figure 4 reports the spectral characterisation of the extinction ratios. The highest extinction for the transmission reached the value of ER_*T*_ = 28.8 dB at 1553.3 nm wavelength while for the reflection the highest value was ER_*R*_ = 18.9 dB at 1549.4 nm. Extinction ratios greater than 15 dB were indeed observed in transmission for a bandwidth of about 14.2 nm. The slight difference in the wavelengths for the maximum values of transmission and reflection extinction ratios can be attributed to the approximated optimisation procedure discussed above, which focuses mainly on one polarisation. Higher values for the extinction ratios might be found with a finer tuning of the interaction length *L*. However, a limitation in the achievable value of the extinction ratio could be given by waveguide non-uniformities, or by slight imbalances of the optical properties between the two waveguides. These may arise e.g. from fluctuations in the power of the waveguide writing laser, or by stress induced on the first waveguide by the writing process of the second one, as they are inscribed sequentially. In particular, even a small, uniform detuning between the propagation constants of the two waveguides forbids^21^ to reach exactly null reflection values; this may explain the fact that the maximum observed values for ER_*R*_ is lower than the maximum observed ER_*T*_.

### Polarisation-insensitive directional couplers

A polarisation-insensitive coupler (PIC) is a device able to split orthogonal polarisations equally. Insensitivity to polarisation is often fundamental in applications that involve polarisation encoding of quantum states, or polarisation-entangled photons, because a polarisation sensitive behaviour would definitely introduce some kind of distinguishability and thus undermine the quality and purity of the quantum states. PICs have been demonstrated recently^17^ with femtosecond laser written waveguides in borosilicate glass substrate, operating in the 800 nm wavelength range. While the component waveguides retained the same optical properties and birefringence of those used elsewhere^15^ for demonstrating partially polarising couplers, there the use of a peculiar three-dimensional geometry enabled an equalisation of the coupling coefficients for the two polarisations, and hence the achievement of polarisation insensitivity. The operation principle of those devices is briefly described as follows. For weakly coupled modes, the coupling coefficients *κ*_*V*,*H*_ are directly proportional to the overlap integrals of the two H- and V-polarised waveguide modes. Since the H- and V-polarised modes present a different ellipticity, if two coupled waveguides lie on a plane tilted with a proper angle with respect to the horizontal, it may be possible to reach a condition for which the two overlap integrals become equal and thus *κ*_*H*_ = *κ*_*V*_.

**Figure 5:**
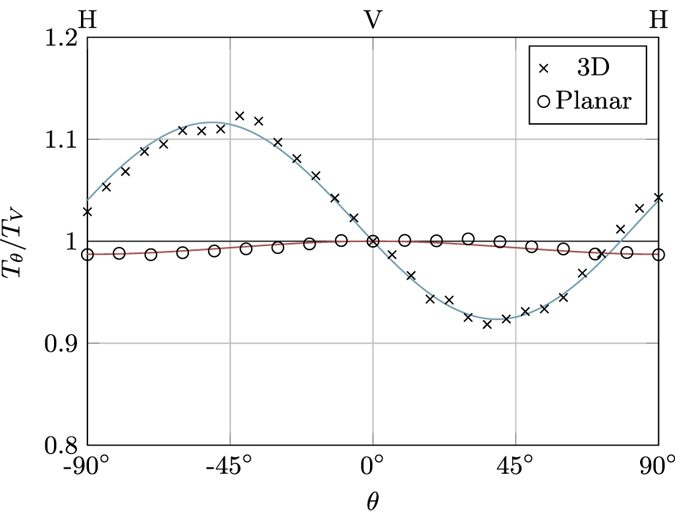
Ratio between the transmission *T*_*θ*_ of a DC, measured by injecting linearly polarised light at a polarisation angle *θ* with respect to the vertical one, and the transmission *T*_*V*_ ≡ *T*_0°_, measured for vertical polarisation. Typical results are reported both for a three-dimensional coupler (crosses), realized with the method of ref. 17, and a planar one (circles), realised with the method discussed in the text. Solid lines are best fits according to Eqs (13) and (14), discussed in the Methods section. Error bars are smaller or comparable to the marker size.

At first, we tried to follow the above approach to devise PICs working at 1550 nm. Indeed, the waveguides used in the present work differ from those reported in refs 15 and 17 only for the higher inscription power (because of the larger operation wavelength, the waveguide size has also to be larger). To this purpose, we fabricated and characterised several DCs with different interaction distances *d* and tilting angles of the coupler plane, observing for some of them the desired equality of splitting ratio for both H- and V-polarised input light. However, a more careful study of their polarisation performance showed the behaviour reported in Fig. 5: light injected with H or V polarisation direction produces an equal splitting ratio, but the splitting ratio changes when the light injected in the coupler is linearly polarised along a more generic angle. Additional measurements also showed that H- or V-polarised input light underwent a slight polarisation rotation while propagating in the couplers. These phenomena can be explained only by assuming that the birefringence axis of the waveguides does not remain unaltered and vertically oriented in the whole directional coupler, but it changes orientation in at least some part of the device (see the Methods section on the formalism). From a physical point of view, the rotation of the birefringence axis in the coupler can be explained by a mechanical stress induced on the first waveguide, within the coupling region, by the inscription of the second waveguide. In fact, Heilmann *et al*.^14^ recently observed similar rotation of the waveguide birefringence axis when inscribing close-by traces at an angle. We observed the same effect in an experiment where an integrated optical circuit fabricated by FLM was used for the spatial multiplexing of orthogonal polarisation light states, for quantum cryptography applications^24^. There, very compact and balanced PICs (*d* = 7 *μ*m) for 850 nm light were inscribed in borosilicate glass using the out-of-plane geometry, which caused small polarisation distortion at the circuit output. Due to the highly demanding tolerances on the output polarisation state required by the specific experiment, a compensation of this non-ideal behaviour was implemented. On the contrary, we never observed significant effects related to this phenomenon in the experiments of refs 15 and 17, as the tiny polarisation rotation induced by the couplers does not alter appreciably the circuit output power distribution.

An alternative route for designing PICs can be individuated by a more careful inspection of Fig. 2a. In fact, one can notice that for shorter and shorter interaction distances *d* the coupling coefficients for the two polarisations tend to become similar. If, by further decreasing *d*, the two coupling coefficients not only become more similar, but also exchange magnitudes, there will be a point in which they cross. Namely, in that point the two coefficients *κ*_*H*_ and *κ*_*V*_ will be equal.

**Figure 6:**
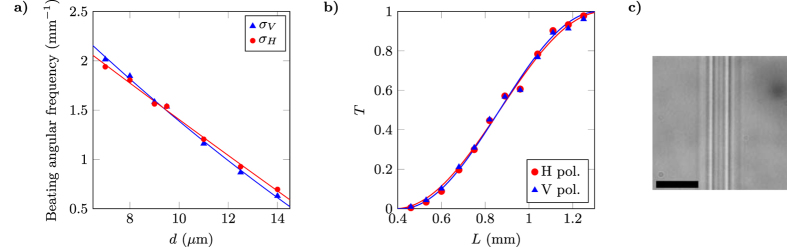
(**a**) Experimentally retrieved values for *σ*_*H*_ (red circles) and *σ*_*V*_ (blue triangles), for inter-waveguide separations comprised between 7 *μ*m and 14 *μ*m. Solid lines are second-order polynomial fits, since for such short interaction distances the exponential approximation (4) is not accurate. Raw experimental data are reported in Supplementary Tables S1 and S2. (**b**) Transmission *T* for PICs built with *d* = 8 *μ*m and *L* = 0.4–1.3 mm, for H- (red circles) and V- (blue triangles) polarised input light. Sinusoidal best fits according to (3) are also plotted. Experimental data plotted in the graph are also reported in Supplementary Table S3. (**c**) Microscope image of the interaction region of one of the PICs with *d* = 8 *μ*m: the two laser-written waveguides have central regions with higher index contrast (whiter in the picture) which are still well apart, but the external modified regions overlap. The scale-bar is 20 *μ*m. Error bars are smaller or comparable to the marker size.

In order to study the validity of this hypothesis, we fabricated and characterised sets of directional couplers with waveguide separations *d* between 7 *μ*m and 14 *μ*m, and lengths *L* spanning from 0 mm up to 1.6 mm. These couplers keep the planar geometry of Fig. 1 and thus differ from the PDCs described previously only for the different geometrical parameters. For each group of devices we retrieved, for the two polarisations, the beating periodicity for the transmission and reflection of the coupler as a function of the length *L*; namely, we could retrieve the quantity *σ* in Eq. (2). We prefer here to describe the power beatings in terms of *σ* and not in terms of *κ* because we cannot exclude the presence of a small detuning between the propagation constants of the two waveguides, as we will discuss below. We observe in Fig. 6a that indeed *σ*_*V*_ = *σ*_*H*_ for *d* $\simeq$ 9 *μ*m, while *σ*_*V*_ > *σ*_*H*_ for shorter distances and vice versa for larger ones.

In practice, to realise a working PIC it is not sufficient to set *d* as close as possible to the distance at which *σ*_*H*_ = *σ*_*V*_. In fact, in the actual device coupling happens both in the central region and in the approaching curved waveguide segments. The overall behaviour results from different coupling contributions occurring at different separations: the central region will likely have to be built with a short distance *d* for which *σ*_*V*_ > *σ*_*H*_, to balance the opposite tendency of the coupling occurring in the curved parts where *σ*_*V*_ < *σ*_*H*_. Figure 6b shows the measured transmission values for DCs with *d* = 8 *μ*m and *L* = 0.4–1.3 mm. The absolute transmission differences for the two polarisations reaches impressively low values down to about 10^−4^, for the device length *L* that most balances the two coupling contributions.

We analysed the performance of these devices for different linear polarisation states at the input. As shown in Fig. 5, the transmission of these DCs remains practically constant for every polarisation angle. The small oscillation with *θ* still discernible in Fig. 5 can be explained by a residual difference in the coupling coefficients of the two polarisations, but is consistent with a birefringence axis that remains fixed and vertically/horizontally oriented throughout the device (see the Methods section). We also checked experimentally that no polarisation rotation occurs when injecting H- or V-polarised light, further verifying that the birefringence axis is not tilted. It is finally important to note that, notwithstanding the very tight separation between the waveguides in the interaction region (see Fig. 6c), we didn’t observe any degradation of the waveguide quality or any additional loss in these DCs with respect to analogous devices with the same waveguide length but larger coupling separation.

Furthermore, the achievement of the PIC condition is not particularly delicate and we observed it quite reproducibly for the optimized value of *d*. In fact, for a coupler as that analyzed in Fig. 6b, one can estimate that a difference |*T*_*V*_ − *T*_*H*_| $\simeq$ 1% between the transmission at the two polarizations would require a difference in the corresponding coupling parameters Δ*σ* $\simeq$ 0.01 mm^−1^ (with respect to the optimum condition). Given the slope of the curves in Fig. 6a this corresponds to an error in setting the distance *d* of about 0.4 *μ*m, which is far larger than the tolerance of our translation stages used for the fabrication of the devices. We have observed experimentally small day-to-day variations of both *σ*_*V*_ and *σ*_*H*_, but without changing their relative magnitude (the reader may compare data reported in Supplementary Tables S2 and S3, regarding two different fabrication session of couplers with *d* = 8 *μ*m): this can be compensated by small adjustments of the optimum *L* for achieving the desired transmission.

The physical reason for such a polarisation insensitivity is likely to be an alteration of the optical properties of the first inscribed waveguide of the coupler, caused by the inscription of the second. In particular, the mechanical stress caused by the second inscribed trace may affect the modal birefringence of the pre-existent waveguide, making it decrease and possibly making it change its sign, for closer and closer waveguides. This effect would be analogous (but opposite in sign) to that reported in ref. 25, where the authors observe a change in the birefringence magnitude when other modification traces are inscribed next to the waveguides, in fused silica substrate. On the contrary, the birefringence of the second inscribed structure would likely remain the same one of the isolated waveguide, since the laser induced material-modification process involves high temperatures that should anneal any pre-existent mechanical stress in the region.

Such alterations of the first waveguide cause a change in its polarisation mode sizes and thus directly influence the coupling coefficients *κ*_*H*_ and *κ*_*V*_. In addition, they may affect the relative detuning between the waveguides, Δ_*H*_ and Δ_*V*_ for the two polarisations. In any case, results in Fig. 6 show that, for a given distance *d*, it is possible to achieve the condition:

8 $${\kappa }_{H}^{2}+{{\rm{\Delta }}}_{H}^{2}/4={\kappa }_{V}^{2}+{{\rm{\Delta }}}_{V}^{2}/4$$

namely, an equalisation of the beating frequencies *σ*_*H*_ and *σ*_*V*_. Since transmission values close to unity are observed experimentally, a possible detuning Δ should actually be much smaller than the coupling coefficient *κ* (see Eq. (2)). This can be reasonable given the very short waveguide separation and high coupling coefficients involved.

## Discussion

We have demonstrated fully polarising DCs, fabricated by femtosecond laser waveguide writing in borosilicate glass, operating in the telecom wavelength range. These devices present extinction ratios between the two polarisations higher than 25 dB at the design wavelength of 1550 nm, maintaining extinction ratios higher than 15 dB in a range of ~14 nm. Additionally, we developed a new architecture that allows to generate highly polarisation insensitive couplers with a planar geometry, using waveguides with the same irradiation parameters as the ones used for the polarising devices. Importantly, this means that it is possible to combine in a single circuit both polarisation dependent and polarisation independent devices operating at the telecom wavelength, by just tuning the geometrical parameters of the components. Improved performances and compactness of the devices might be pursued by varying the irradiation parameters used for waveguide writing. Thermal annealing techniques^26^ may also be investigated to ameliorate waveguide uniformity, enhance the achievable extinction ratios of PDCs, and at the same time decrease both propagation losses and coupling efficiency with fibers.

**Figure 7:**
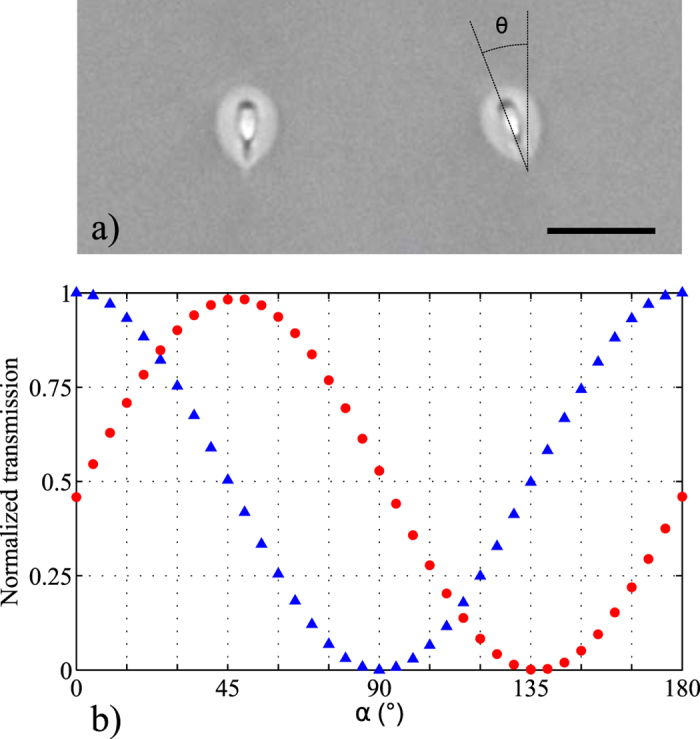
(**a**) Cross section of optical waveguides fabricated in EAGLE glass with the technique presented in ref. 16, here yielding single-mode operation at 1550 nm wavelength. The waveguide on the left is fabricated with vertical optical axis while the one on the right presents an optical axis tilted by an angle *θ* $\simeq$ 23°. Scale-bar is 20 *μ*m. Both waveguides are 25 mm long. (**b**) Polarisation analysis of the output state when launching H-polarised light at the waveguide input, for the vertical-axis waveguide (blue triangles) and for the tilted-axis one (red circles). To perform the measurement, a rotatable polariser at an angle *α* is placed at the waveguide output and the transmitted power is recorded as a function of *α*. Data have been normalised with respect to the power recorded at *α* = 0 for the vertical-axis waveguide. Error bars are smaller or comparable to the marker size.

These results become even more significant, as we have proven experimentally that the same technique presented in ref. 16 for writing waveguides with rotated optical axis for arbitrary polarisation manipulation can be straightforwardly extended to telecom C-band devices. In particular, we show in Fig. 7 the results for a waveguide with tilted birefringent axis, tailored for acting as an integrated half-waveplate operating at 1550 nm wavelength, compared to a waveguide with vertical axis. Figure 7a compares the cross sections as visible with an optical microscope. The waveguide brefringence was characterised using the method of ref. 5: the direction of the birefringence axis is found coincident with the symmetry axis of the waveguide (*θ* $\simeq$ 23°), while the measured birefringence values are *b*_1_ = 2.7 · 10^−5^ for the vertical-axis waveguide and *b*_2_ = 3.1 · 10^−5^ for the tilted-axis one. To confirm the waveplate-like behaviour of the tilted-axis waveguide we launched H-polarised light in the waveguide input and we placed a rotatable linear polariser at the output. Figure 7b reports the normalised transmitted power, measured after the polariser, as a function of its rotation angle *α*: in the case of the tilted-axis waveguide (red circles) power oscillates sinusoidally with practically unitary maximum at about 45° and null minimum at 135°. This indicates a linearly polarized output states with 45° polarisation direction, consistently with the expected behaviour for a half-waveplate tilted at about 22.5°. The same measurement performed on the vertical-axis waveguide is also reported for comparison (blue triangles), showing that the H-polarized input state propagates unperturbed.

The results presented in this work extend in the telecom wavelength band the capability of femtosecond laser written circuits to handle and manipulate the polarisation degree of freedom, which was reported in the recent years in the 800 nm wavelength region^5, 15, 17^. This paves the way to the integrated manipulation of polarisation encoded photonic qubits and polarisation entanglement at this interesting wavelength range. Notably, and differently from other integrated platform such as silicon photonic circuits, femtosecond laser written waveguides present very good mode-matching and easy interfacing with standard optical fibers. These features could thus promote the application of integrated-optics technology, with all its advantages in terms of stability and compactness, to practical demonstrations of quantum communication protocols within standard fiber networks. In addition, the control of polarisation, combined with the phase stability intrinsic to waveguide circuits, may open further perspectives to integrated quantum optics experiments with hyper-entangled photons^27^.

## Methods

### Waveguides fabrication and characterisation

The devices were fabricated using a femtosecond Yb:KYW cavity-dumped mode-locked oscillator, emitting pulses of 300 fs at 1 MHz repetition rate, with a wavelength *λ* = 1030 nm. The laser beam was focused 170 *μ*m beneath the surface of Corning Eagle2000 alumino-borosilicate glass, using a 50× microscope objective of 0.6 NA. The translation of the sample was performed by computer controlled air-bearing stages (Aerotech FiberGLIDE 3D). Irradiation parameters for the inscription of single-mode waveguide for 1550 nm operation wavelength were 370 nJ pulse energy and 40 mm s^−1^ translation speed. Coupling losses to standard single mode fibres at 1550 nm are 0.4 dB, estimated by evaluating numerically the overlap integral between the measured mode profile of the waveguide and that of a fibre. Measured propagation losses are 0.3 dB cm^−1^. The radius of curvature employed for the segments of the DCs was 90 mm, giving additional bending losses of 0.4 dB cm^−1^. To measure waveguide birefringence we adopted the method described in the Supplemental Material of ref. 5: namely, we injected light with different polarisation states in the waveguides and we characterised the polarisation states at the output, then best fitting the birefringence value that provided the observed transformation.

### Characterization of the DCs

To characterize the DCs, laser light (V- or H- polarised) was launched in either input port, using a 0.25 NA objective. Output light was collimated by a 0.5 NA objective. The output power was measured with an optical power meter for both outputs, allowing to evaluate the DC’s transmission and reflection (*T* and *R*) according to Eq. (1). The power meter sensitivity and resolution is below 1 *μ*W and, in our characterisation measurements, the typical total power at the output of a DC was in the mW scale. In addition, by placing the power meter head far enough from the objective, the impact of background light (light not coupled in the waveguide and propagating in the glass substrate) was reduced to less than 1/100 of the total signal. Therefore, the measured values have at least 2 or 3 reliable significant figures and, if reported in the graphs, error bars would be smaller than the employed marker size.

### Generic polarisation description of two-waveguide devices

A generic linear integrated-optics device having two input waveguides and two output waveguides, where two polarisation modes are considered for each distinct input/output port, operates as a linear transformation from four input optical modes to four output optical modes. In the lossless case, this transformation is unitary. If the waveguides at the input and output are birefringent with a vertical axis, the two polarisation modes can be assumed as linearly polarised modes, with H or V orientation.

In detail, the operation of the device can be described as a unitary matrix:

9 $$U=[\begin{array}{cccc}{a}_{1} & {a}_{2} & {b}_{1} & {b}_{2}\\ {a}_{3} & {a}_{4} & {b}_{3} & {b}_{4}\\ {c}_{1} & {c}_{2} & {d}_{1} & {d}_{2}\\ {c}_{3} & {c}_{4} & {d}_{3} & {d}_{4}\end{array}]$$

applied to the vector of the mode field amplitudes:

10 $$\bar{E}=[\begin{array}{c}{H}_{1}\\ {H}_{2}\\ {V}_{1}\\ {V}_{2}\end{array}]$$

where *H*_*i*_ and *V*_*i*_ are the modes of the port *i*, respectively H- or V- polarised. In a quantum description, the same formalism can be used if the elements of the vector (10) are considered as the mode annihilation operators.

It can be noted the terms *b*_*j*_ and *c*_*j*_ in the matrix (9) makes the H- and V- polarised modes interact and exchange optical power. All those terms should then be vanishing if the birefringence axis of the waveguides remain uniform and vertically oriented in the whole device: in fact the H and V polarised modes remain separated and never exchange power. In that case, the device behaves as two independent couplers operating on the H and V polarisation respectively:

11 $${U}_{c}=[\begin{array}{cccc}{r}_{H} & \iota \,{t}_{H} & 0 & 0\\ \iota \,{t}_{H}^{\ast } & {r}_{H}^{\ast } & 0 & 0\\ 0 & 0 & {r}_{V}{e}^{\iota \beta } & \iota \,{t}_{V}{e}^{\iota \beta }\\ 0 & 0 & \iota \,{t}_{V}^{\ast }{e}^{\iota \beta } & {r}_{V}^{\ast }{e}^{\iota \beta }\end{array}]$$

where *r*_*H*_, *r*_*V*_, *t*_*V*_ and *t*_*H*_ are complex coefficients, *β* is a phase term which accounts for birefringence effects (which delay one polarisation more than the other after a propagation through the whole device), and global phase terms on the whole matrix are neglected. If such device physically correspond to a DC as the one in Fig. 1, built of two identical waveguides, one can write more precisely *r*_*H*_ = cos(*κ*_*H*_ *L* + *ϕ*_*H*_), *t*_*H*_ = sin(*κ*_*H*_ *L* + *ϕ*_*H*_), *r*_*V*_ = cos(*κ*_*V*_ *L* + *ϕ*_*V*_), *t*_*V*_ = sin(*κ*_*V*_ *L* + *ϕ*_*V*_), using notation consistently with the one used in the text.

### Coupler operation for generic linearly polarised input light

Linearly polarised coherent light at a generic orientation *θ* with respect to the V axis, injected in one input port of the coupler (e.g., input port 1), is described by a vector written as:

12 $$\bar{E}={E}_{0}\cdot [\begin{array}{c}\sin \,\theta \\ 0\\ \cos \,\theta \\ 0\end{array}]$$

where *E*_0_ is the input field amplitude and the formalism of Eqs (9 and 10) is adopted.

It is not difficult to show that, in the case of generic device described by a matrix (9), the power transmission at the output 2, defined as in (1), takes the form:

13 $$T={C}_{1}+{C}_{2}\,\cos \,2\theta +{C}_{3}\,\sin \,2\theta $$

where ${C}_{1}=\tfrac{1}{2}\,({|{a}_{3}|}^{2}+{|{b}_{3}|}^{2}+{|{c}_{3}|}^{2}+{|{d}_{3}|}^{2})$, ${C}_{2}=\tfrac{1}{2}\,({|{a}_{3}|}^{2}+{|{b}_{3}|}^{2}-{|{c}_{3}|}^{2}-{|{d}_{3}|}^{2})$ and ${C}_{3}=\Re \{{a}_{3}{b}_{3}^{\ast }+{c}_{3}{d}_{3}^{\ast }\}$. Namely, the power transmission oscillates generically with the polarisation orientation *θ*, with a periodicity of 180°.

On the other hand, if we consider a coupler with fixed vertical birefringence axis, whose matrix takes the form (11), the coefficient *C*_3_ in (13) vanishes, and *T* becomes:

14 $$T=\frac{1}{2}[{|{t}_{H}|}^{2}+{|{t}_{V}|}^{2}+({|{t}_{V}|}^{2}-{|{t}_{H}|}^{2})\,\cos \,2\theta ].$$

Actually, *T* oscillates sinusoidally between the transmission values observed for pure H and V polarisations, having the maxima or minima exactly in *θ* = 0° and *θ* = ±90°.

### Data availability

The datasets generated during and/or analysed during the current study are available from the corresponding author on reasonable request.

## Electronic supplementary material

Supplementary data

## References

[1] Kenichi, I. & Kokubun, Y. Encyclopedic Handbook of Integrated Optics. CRC Press (2005).

[2] Politi A, Cryan MJ, Rarity JG, Yu S, O’Brien JL (2008). Silica-on-silicon waveguide quantum circuits. Science.

[3] Silverstone JW (2014). On-chip quantum interference between silicon photon-pair sources. Nat Photon.

[4] Marshall GD (2009). Laser written waveguide photonic quantum circuits. Opt Express.

[5] Sansoni L (2010). Polarisation entangled state measurement on a chip. Phys. Rev. Lett..

[6] Meany T (2015). Laser written circuits for quantum photonics. Laser Photon Rev.

[7] Crespi A (2016). Suppression law of quantum states in a 3D photonic fast Fourier transform chip. Nat Commun.

[8] Bricchi E, Klappauf BG, Kazansky PG (2004). Form birefringence and negative index change created by femtosecond direct writing in transparent materials. Opt Lett.

[9] Bhardwaj VR (2004). Stress in femtosecond-laser-written waveguides in fused silica. Opt Lett.

[10] Kapron F, Borrelli N, Keck D (1972). Birefringence in dielectric optical waveguides. IEEE J Quant Electron.

[11] Snyder AW, Zheng X (1986). Optical fibers of arbitrary cross sections. J Opt Soc Am A.

[12] Fernandes LA, Grenier JR, Herman PR, Aitchison JS, Marques PVS (2011). Femtosecond laser fabrication of birefringent directional couplers as polarisation beam splitters in fused silica. Opt Express.

[13] Fernandes LA, Grenier JR, Herman PR, Aitchison JS, Marques PVS (2011). Femtosecond laser writing of waveguide retarders in fused silica for polarisation control in optical circuits. Opt Express.

[14] Heilmann R, Gräfe M, Nolte S, Szameit A (2014). Arbitrary photonic wave plate operations on chip: Realising Hadamard, Pauli-X, and rotation gates for polarisation qubits. Sci Rep.

[15] Crespi A (2011). Integrated photonic quantum gates for polarisation qubits. Nat Commun.

[16] Corrielli G (2014). Rotated waveplates in integrated waveguide optics. Nat Commun.

[17] Sansoni L (2012). Two-Particle Bosonic-Fermionic Quantum Walk via Integrated Photonics. Phys Rev Lett.

[18] Stucki D, Gisin N, Guinnard O, Ribordy G, Zbinden H (2002). Quantum key distribution over 67 km with a plug-and-play system. New J Phys.

[19] Serafini A, Mancini S, Bose S (2006). Distributed Quantum Computation via Optical Fibers. Phys Rev Lett.

[20] Boschi D, Branca S, De Martini F, Hardy L, Popescu S (1998). Experimental Realization of Teleporting an Unknown Pure Quantum State via Dual Classical and Einstein-Podolsky-Rosen Channels. Phys Rev Lett.

[21] Yariv A (1973). Coupled-mode theory for guided-wave optics. IEEE J Quant Electron.

[22] Huang WP (1994). Coupled-mode theory for optical waveguides: an overview. J Opt Soc Am A.

[23] Szameit A, Dreisow F, Pertsch T, Nolte S, Tünnermann A (2007). Control of directional evanescent coupling in fs laser written waveguides. Opt Express.

[24] Vest G (2015). Design and evaluation of a handheld quantum key distribution sender module. IEEE J Sel Top Quantum Electron.

[25] Fernandes LA, Grenier JR, Herman PR, Aitchison JS, Marques PVS (2012). Stress induced birefringence tuning in femtosecond laser fabricated waveguides in fused silica. Opt Express.

[26] Arriola A (2013). Low bend loss waveguides enable compact, efficient 3D photonic chips. Opt Express.

[27] Ciampini MA (2016). Path-polarization hyperentangled and cluster states of photons on a chip. Light Sci Appl.

