# Supplementary material for: Geometrically-controlled polarisation processing in femtosecond-laser-written photonic circuits
Source: Sci Rep. 2017 Sep 12;7:11342. doi: 10.1038/s41598-017-09462-0 (PMC5596026; doi:10.1038/s41598-017-09462-0)
Supplement: Supplementary file 1 — Supplementary data [file 41598_2017_9462_MOESM1_ESM.pdf]

Geometrically-controlled polarisation processing in femtosecond-laser-written photonic circuits — Supplementary Information —

Ioannis Pitsios,[1,2] Farid Samara,[2,*] Giacomo Corrielli,[1,2] Andrea Crespi,[2,1] and Roberto Osellame[1,2,†]

[1]*Istituto di Fotonica e Nanotecnologie - Consiglio Nazionale delle Ricerche (IFN-CNR), p.za Leonardo da Vinci 32, 20133 Milano, Italy*

[2]*Dipartimento di Fisica - Politecnico di Milano, p.za Leonardo da Vinci 32, 20133 Milano, Italy*

d (μm)	σ_V (mm^{-1})	σ_H (mm^{-1})
7.0	2.01	1.94
8.0	1.85	1.80
9.0	1.58	1.56
9.5	1.54	1.55
11.0	1.15	1.22
12.5	0.87	0.96
14.0	0.62	0.71

Supplementary Table S1. Experimental values of σ_V and σ_H, as plotted in Fig. 6a in the Main Text, as a function of the waveguide separation d in a directional coupler (DC). To retrieve these values we fabricated several sets of DCs scanning the interaction length L for each different d, we characterized the DC transmission for both input polarisations (data reported in Supplementary Table S2), and then we performed a best fit of Eq. (2) in the Main Text. Experimental uncertainty of the reported values is $\Delta\sigma = 0.03$ mm^{-1}.

$d = 7.0$ μm			$d = 8.0$ μm			$d = 9.0$ μm		
L (mm)	T_V	T_H	L (mm)	T_V	T_H	L (mm)	T_V	T_H
0.00	0.18	0.18	0.00	0.44	0.47	0.00	0.66	0.65
0.23	0.00	0.00	0.23	0.05	0.06	0.23	0.27	0.28
0.46	0.22	0.19	0.46	0.05	0.04	0.46	0.02	0.03
0.70	0.68	0.62	0.70	0.44	0.43	0.70	0.06	0.05
0.93	0.99	0.97	0.93	0.90	0.88	0.93	0.36	0.34
1.16	0.89	0.94	1.16	0.97	0.99	1.16	0.73	0.71
1.39	0.49	0.60	1.39	0.65	0.70	1.39	0.99	0.97

$d = 9.5$ μm			$d = 11.0$ μm			$d = 12.5$ μm			$d = 14.0$ μm		
L (mm)	T_V	T_H	L (mm)	T_V	T_H	L (mm)	T_V	T_H	L (mm)	T_V	T_H
0.00	0.74	0.75	0.00	0.89	0.90	0.00	0.86	0.88	0.00	0.41	0.42
0.20	0.45	0.46	0.20	0.73	0.72	0.20	0.97	0.98	0.20	0.54	0.56
0.40	0.17	0.17	0.40	0.49	0.46	0.40	0.99	0.99	0.40	0.65	0.69
0.60	0.01	0.01	0.60	0.28	0.24	0.60	0.99	0.96	0.60	0.78	0.81
0.80	0.03	0.04	0.80	0.10	0.07	0.80	0.90	0.88	0.80	0.87	0.92
1.00	0.22	0.23	1.00	0.01	0.00	1.00	0.76	0.72	1.00	0.93	0.96
1.20	0.51	0.52	1.20	0.02	0.05	1.20	0.61	0.55	1.20	0.97	0.99
1.40	0.80	0.81	1.40	0.13	0.20	1.40	0.44	0.37	1.40	1.00	0.98
			1.60	0.32	0.43	1.60	0.27	0.20	1.60	0.98	0.92

Supplementary Table S2. Experimental values of the transmission T_V and T_H (i.e., measured for V and H input polarisations respectively) in directional couplers with different interwaveguide distance d and length of the interaction region L. Measurement uncertainty in the reported values is $\Delta T = 0.01$.

L (mm)	T_V	T_H
0.46	0.00	0.01
0.53	0.03	0.04
0.60	0.09	0.10
0.68	0.20	0.21
0.75	0.30	0.31
0.82	0.45	0.45
0.89	0.57	0.57
0.96	0.61	0.60
1.04	0.78	0.77
1.11	0.90	0.89
1.18	0.93	0.91
1.25	0.98	0.96

Supplementary Table S3. The power transmission for PICs built with d = 8 μm and L = 0.4 - 1.3 mm, for V and H polarised input light, as plotted in Fig. 6b of the Main Text. Measurement uncertainty in the reported values is $\Delta T = 0.01$.
